# Immobilization of endo-xylanase from *Thermomyces lanuginosus* PC7S1T for selective production of xylooligosaccharides from agricultural residues

**DOI:** 10.1007/s13205-026-04857-1

**Published:** 2026-05-21

**Authors:** Laura Camila Wagner Rodio, Maria Eduarda Carboniéri, Tatiane Sayuri Inagaki, Diandra de Andrades, Maria de Lourdes Teixeira de Moraes Polizeli, Paula Daniela Helfenstein Rother, Marco Antônio Záchia Ayub, José Luis da Conceição Silva, Alexandre Maller, Thaís Duarte Bifano, Rita de Cássia Garcia Simão, Marina Kimiko Kadowaki

**Affiliations:** 1 Center of Medical Sciences and Pharmaceutical, Western Paraná State University, Rua Universitária 2069, Cascavel, PR ZC 85819-110 Brazil; 2 Department of Biology, Faculty of Philosophy, Sciences and Letters of Ribeirão Preto, University of São Paulo, Ribeirão Preto, SP 14040-901 Brazil; 3 Biotechnology, Bioprocess, and Biocatalysis Group, Food Science and Technology Institute, Federal University of Rio Grande Do Sul, Av. Bento Gonçalves 9500, PO Box 15090, Porto Alegre, RS ZC 91501-970 Brazil

**Keywords:** Xylooligosaccharides, Immobilized xylanase, Magnetic chitosan, Agricultural residues, Prebiotics

## Abstract

Xylooligosaccharides (XOS) were produced from xylan-rich agricultural residues using endo-xylanase from *Thermomyces lanuginosus* PC7S1T immobilized on magnetic chitosan beads. The immobilized enzyme showed high immobilization yield (90.7%), efficiency (73.7%), and activity recovery (66.8%). Enhanced stability was observed, with 94% residual activity after 50 d at 4 °C and 54% after 10 reuse cycles, along with improved thermal (70–75 °C) and pH stability (pH 6.5). Immobilization altered the product profile, favoring xylotriose (X3) and xylotetraose (X4), whereas the free enzyme predominantly produced xylobiose (X2), highlighting its potential for prebiotic applications. Scanning electron microscopy confirmed effective substrate depolymerization. Toxicity assays using *Artemia salina* showed no significant differences in survival between XOS produced by free and immobilized enzymes (*p* > 0.05), indicating low acute toxicity. Overall, these results reveal the potential of this immobilized system for sustainable and scalable production of prebiotic XOS from renewable biomass.

## Introduction

Xylooligosaccharides (XOS) are short-chain β-(1 → 4)-linked xylose oligomers (degree of polymerization, 2–10) that are widely recognized for their prebiotic properties, particularly their ability to selectively stimulate beneficial gut microbiota and promote associated health benefits (Santibáñez et al. 2021). Because of these functional attributes, XOS have become increasingly relevant in food, nutraceutical, and animal feed applications (Kaushal et al. 2021).

Xylan-rich agricultural residues, such as corn cobs, wheat straw, and sugarcane bagasse, represent abundant and renewable substrates for XOS production. Their enzymatic valorization supports circular bioeconomy strategies by converting lignocellulosic waste into value-added bioproducts (Huang et al. 2018; Cartaxo da Costa Urtiga et al. 2020).

Endo-β-1,4-xylanases (EC 3.2.1.8) catalyze the internal cleavage of xylan chains, generating oligomers such as xylobiose (X2), xylotriose (X3), and xylotetraose (X4) (Kaushal et al. 2021). Among microbial enzymes, GH11 xylanases from *Thermomyces lanuginosus* are particularly attractive because of their thermostability, substrate specificity, and industrial robustness (Shi et al. 2019). In addition, enzymatic hydrolysis provides milder reaction conditions and greater product selectivity compared with those of chemical methods (Romero-Fernández et al. 2018).

Despite these advantages, the industrial application of soluble xylanases remains limited by low operational stability, lack of catalyst recovery, and poor reusability (Guisan et al. 2020; Borzouee et al. 2021). Therefore, enzyme immobilization has been widely explored as a strategy to enhance stability and enable enzyme reuse. Magnetic chitosan-based supports, particularly those activated with glutaraldehyde, have been frequently used for xylanase immobilization, with studies focusing primarily on improvements in thermal stability and operational performance (Hamzah et al. 2019).

However, the impact of enzyme immobilization on XOS product distribution and selective oligomer production remains poorly understood. In particular, limited attention has been given to the enrichment of specific oligomers such as X3 and X4, which are associated with enhanced prebiotic effects (Santibáñez et al. 2021). Moreover, studies integrating selective XOS production from agricultural residues, operational stability of immobilized enzymes, and toxicity assessment of the resulting products are still scarce.

In the present study, a thermostable GH11 endo-xylanase from *T. lanuginosus* PC7S1T was immobilized onto magnetic chitosan beads to develop a reusable biocatalytic system for selective XOS production from agricultural residues. The effects of immobilization on catalytic performance, oligomer distribution, operational stability, and product safety were systematically evaluated, with the aim of advancing sustainable and targeted prebiotic production.

## Materials and methods

### Fungus maintenance

*T. lanuginosus* PC7S1T, previously isolated from Atlantic Forest soil in western Paraná, Brazil, was taxonomically identified by Corrêa et al. (2016). Its sequence is available in the National Center for Biotechnology Information (NCBI) database under accession number KJ934703.1. The organism was routinely maintained on potato dextrose agar slants at 4 °C.

### Enzyme production and purification

The enzyme extract was obtained through submerged cultivation in Czapek medium supplemented with 1.5% (w/v) corn straw and inoculated with a spore suspension (10⁷ spores/mL). After 4 d of incubation at 42 °C under agitation (120 rpm), the culture was filtered under vacuum through Whatman filter paper to obtain the crude extract. The filtrate was dialyzed against deionized water for 18 h at 7 °C. Next, the crude extract was centrifuged at 2150 × g for 10 min at 4 °C, and the supernatant was applied to a diethylaminoethyl (DEAE)-Sepharose column (2 × 20 cm) equilibrated with 0.02 M Tris–HCl buffer (pH 7.2), according to the method reported by Della Torre et al. (2023). Bound proteins were eluted using a linear NaCl gradient (0.05–1.0 M), and fractions exhibiting xylanase activity were pooled and dialyzed against distilled water for 18 h at 7 °C. The purification step was repeated using a second DEAE-Sepharose column (2 × 20 cm). Active fractions were pooled, dialyzed, and concentrated by lyophilization. The concentrated enzyme was subsequently applied to a Sephadex G-75 gel filtration column (2 × 60 cm), pre-equilibrated with 0.05 M sodium phosphate buffer (pH 6.5), and eluted at a flow rate of 0.5 mL/min. Fractions showing xylanase activity were pooled, dialyzed, and used for immobilization and further characterization.

### Enzyme and protein assays

Xylanase activity was determined by mixing 25 µL of 1% (w/v) beechwood xylan in 0.05 M sodium phosphate buffer (pH 6.5) with 25 µL of *T. lanuginosus* endo-xylanase, followed by incubation at 65 °C for 15 min. The reaction was stopped by adding 50 µL of 3,5-dinitrosalicylic acid reagent, and the released reducing sugars were quantified according to the method reported by Miller (1959), using D-xylose as the standard. One unit of enzyme activity (U) was defined as the amount of enzyme required to release 1 µmol of reducing sugar per minute under the assay conditions.

Protein concentration was determined using the Bradford method (1976) with bovine serum albumin as the standard. Protein content during purification was monitored by measuring absorbance at 280 nm using a spectrophotometer.

### Preparation of magnetic chitosan beads

Magnetic particles were prepared by the co-precipitation method according to the process reported by Maity and Agrawal (2007) and Sojitra et al. (2016). Briefly, an aqueous solution containing Fe^2^⁺ (0.2 M) and Fe^3^⁺ (0.4 M) chlorides (molar ratio 1:2) was prepared in 45 mL of sonicated distilled water and stirred at 1000 rpm at 70 °C for 30 min. Subsequently, 25% (w/v) sodium hydroxide was added dropwise to induce precipitation, resulting in the formation of black magnetic particles. The precipitate was separated using a neodymium magnet, washed thoroughly with distilled water, and dried at 40 ± 2 °C overnight.

Chitosan (Sigma-Aldrich, St. Louis, MO, USA) was incorporated into the magnetic particles by dissolving 2.5 g of chitosan in 2.5% (v/v) acetic acid. The solution was then added dropwise to a coagulation bath containing 40% (v/v) ethanol and 1.0 mol L⁻^1^ NaOH using a peristaltic pump equipped with a 1.5 mm outlet. The resulting magnetic chitosan beads were maintained in an ice bath for 30 min, filtered, and washed with distilled water until neutral pH was reached. The beads were then dried and stored at 4 °C until further use.

### Enzyme immobilization

Magnetic chitosan beads were activated by incubation with 1% (v/v) glutaraldehyde under continuous agitation for 18 h, followed by thorough washing with distilled water to remove excess reagent. Endo-xylanase (100 U) in 0.01 M sodium phosphate buffer (pH 7.0) was then added to the activated beads and gently agitated for 6 h to enable immobilization. After incubation, the immobilized enzyme was washed with distilled water to remove unbound protein and stored at 4 °C in distilled water until further use.

Immobilization performance was evaluated based on the parameters described by Sheldon and van Pelt (2013):

$${\mathrm{Immobilization}}\;{\mathrm{yield}}\left( \% \right) = \left( {{\mathrm{immobilized}}\;{\mathrm{activity}}/{\mathrm{initial}}\;{\mathrm{activity}}} \right) \times {1}00$$

$${\mathrm{Immobilization}}\;{\mathrm{efficiency}}\left( \% \right) = \left( {{\mathrm{observed}}\;{\mathrm{activity}}/{\mathrm{immobilized}}\;{\mathrm{activity}}} \right) \times {1}00$$

$${\mathrm{Activity}}\;{\mathrm{recovery}}\left( \% \right) = \left( {{\mathrm{observed}}\;{\mathrm{activity}}/{\mathrm{initial}}\;{\mathrm{activity}}} \right) \times {1}00$$

### Reusability and storage stability of immobilized endo-xylanase

Reusability was evaluated by incubating the immobilized enzyme with 1% (w/v) beechwood xylan in 0.05 M sodium phosphate buffer (pH 6.5) at 65 °C for 15 min. After each cycle, aliquots were collected to determine the concentration of reducing sugars using the Miller (1959) method. To initiate a new cycle, the immobilized enzyme was washed thrice with 0.05 M sodium phosphate buffer (pH 6.5), and the assay was repeated for a total of 10 cycles.

Storage stability was assessed by storing the immobilized enzyme in distilled water at 4 °C and measuring residual enzymatic activity at 7 d intervals over a period of 50 d.

### a. Effect of temperature, pH, and stability on immobilized xylanase activity

The effect of temperature on the activity of free and immobilized endo-xylanase was evaluated at 40, 50, 60, 70, 75, 80, and 90 °C. Thermal stability was assessed by incubating free and immobilized enzymes in the absence of substrate for up to 5 h at 70 °C and 75 °C.

The influence of pH on enzyme activity was determined using 0.1 M McIlvaine buffer at pH 3.0, 4.0, 5.0, 6.0, 6.5, and 7.0. pH stability was evaluated by incubating the enzyme in McIlvaine buffer at pH 6.5, 7.0, and 7.5 for up to 5 h at 4 °C. Enzymatic activity was expressed as residual xylanase activity (%).

### Pretreatment and crude xylan extraction from agricultural residues

Agricultural residues, including corn straw and corn cob (*Zea mays*), sugarcane bagasse (*Saccharum officinarum*), and sorghum biomass (leaves and stalks of *Sorghum bicolor*), were obtained from agro-industries in the Cascavel region, Paraná State, Brazil.

Alkaline–oxidative pretreatment was performed to promote lignin disruption and facilitate xylan solubilization, according to a method adapted from Freitas et al. (2021). The residues were treated with an alkaline hydrogen peroxide solution containing 5% (v/v) H₂O₂ and 2% (w/v) NaOH at a solid-to-liquid ratio of 1:20 (w/v). The suspensions were incubated at 150 rpm for 4 h at 25 °C, followed by overnight incubation at 80 °C. The treated material was then filtered under vacuum using a Büchner funnel to obtain a liquid fraction enriched in solubilized xylan.

The pH of the filtrate was adjusted to 6.0 using HCl, and the solution was concentrated at 50 °C to approximately one-third of its initial volume. Crude xylan was precipitated by the addition of three volumes of 96% (v/v) ethanol and incubated at 7 °C for 24 h (Nascimento et al. 2022). The precipitate was recovered through filtration, dried at 50 °C, and stored until further use.

### Production and analysis of XOS

Crude xylan extracted from agricultural residues (sorghum, sugarcane bagasse, corn straw, and corn cob) was suspended in 0.05 M phosphate buffer (pH 6.5) at 2% (w/v). For enzymatic hydrolysis, free or immobilized endo-xylanase (30 U) was added, and the reaction mixture was diluted 1:1 with buffer/enzyme solution, resulting in a final crude xylan concentration of 10 g/L. Reactions were carried out at 65 °C under constant agitation (200 rpm) for 48 h. The reaction was stopped by heating in a boiling water bath for 5 min. Control samples containing crude xylan without enzyme were processed under identical conditions.

XOS produced from crude xylan were quantified using high-performance liquid chromatography (Shimadzu, Tokyo, Japan) equipped with a refractive index detector and an Aminex HPX-87C column (300 mm × 7.8 mm). Ultra-pure water was used as the mobile phase at a flow rate of 0.6 mL/min, and the column temperature was maintained at 85 °C. Xylose (X1), xylobiose (X2), xylotriose (X3), xylotetraose (X4), and xylopentaose (X5) were used as external standards to construct calibration curves. Total XOS concentration was calculated as the sum of X2–X5.

XOS conversion from crude xylan was calculated based on a final substrate concentration of 10 g/L after dilution. XOS concentrations were corrected to anhydrous xylan equivalents using a factor of 0.88 (132/150). Conversion (%) was calculated as the ratio of corrected XOS concentration to the initial crude xylan concentration × 100 (Wang et al. 2018).

### Scanning electron microscopy (SEM) analysis of crude xylan surfaces after enzymatic hydrolysis

The effect of enzymatic hydrolysis by the immobilized enzyme on crude xylan extracted from agricultural residues was analyzed using SEM. The samples were mounted on aluminum stubs using double-sided carbon tape and sputter-coated with a 20-nm gold layer in a DENTON Vacuum DESK V evaporator. Imaging was performed using a TESCAN VEGA3 40XVP SEM operated at 25 kV. Untreated crude xylan samples were used as controls.

### Toxicity analysis of XOS produced from crude xylan by free and immobilized endo-xylanase in *Artemia salina*

*A. salina* cysts (10 mg) were incubated in saline solution (15 g/L NaCl in distilled water) at 25 °C for 48 h to enable hatching of nauplii, following the manufacturer’s instructions. Toxicity assays were performed using five nauplii per test tube containing 5 mL of saline solution supplemented with XOS (1 mg/mL) obtained from the enzymatic hydrolysis of crude xylan by free or immobilized endo-xylanase. Control groups consisted of nauplii maintained in saline solution without XOS. All treatments were conducted in triplicate and incubated at 25 °C. Survival was assessed after 24 h by observing antenna movement within 10 s after gentle agitation, as described by Pecoraro et al. (2021). The survival rate (%) was expressed relative to the control group, which showed 100% survival in the absence of XOS.

### Statistical analysis

All experiments were performed at least in duplicate, and results are expressed as the mean ± standard deviation. Data listed in Table 1 were analyzed using GraphPad Prism (GraphPad Software, San Diego, CA, USA). Comparisons between free and immobilized enzymes for each crude xylan source were performed using Student’s *t*-test (*p* < 0.005; **p < 0.0005). Data listed in Table 2 (*A. salina* assays) were analyzed using TIBCO Statistica® (Version 14.1.0.8; TIBCO Software Inc., Palo Alto, CA, USA) using one-way ANOVA followed by Tukey’s test (*p* < 0.05). Lowercase letters indicate differences among substrates, and uppercase letters indicate no differences between enzyme systems for the same substrate.

**Table 1:** Xylooligosaccharide (XOS) production from crude xylan by free and immobilized endo-xylanase

Source of hemicellulose	Enzyme system	X2 (g L^−1^)	X3 (g L^−1^)	X4 (g L^−1^)	X5 (g L^−1^)	Total XOS (g L^−1^)	Conversion (%)
Sorghum biomass	Free	0.64 ± 0.03^***^	0.488 + 0.01	0.48 ± 0.03	0 ± 0.00	1.624	14.3
	Immobilized	0.11 ± 0.00^**^	0.41 ± 0.02	0.47 ± 0.01	0 ± 0.00	1.004	8.83
Sugarcane bagasse	Free	0.66 ± 0.00^***^	0.53 ± 0.03	0.47 ± 0.03	0 ± 0.00	1.672	14.71
	Immobilized	0.19 ± 0.01^***^	0.41 ± 0.01	0.46 ± 0.02	0.11 ± 0.00^**^	1.198	10.54
Corn straw	Free	0.69 ± 0.10^***^	0.48 ± 0.01	0.42 ± 0.03	0 ± 0.00	1.685	14.83
	Immobilized	0.11 ± 0.00^***^	0.37 ± 0.03	0.42 ± 0.03	0 ± 0.00	0.802	7.06
Corn cob	Free	0.51 ± 0.02^***^	0.45 ± 0.02	0.43 ± 0.02	0 ± 0.00	1.402	12.34
	Immobilized	0.10 ± 0.00^***^	0.44 ± 0.04	0.47 ± 0.02	0 ± 0.00	1.026	9.03

^*^Data are expressed as mean ± standard deviation (SD) from two independent experiments (n = 2). X2–X5 correspond to xylobiose, xylotriose, xylotetraose, and xylopentaose, respectively. FE: free enzyme; IE: immobilized enzyme. Statistical analysis was performed using Student’s t-test (GraphPad Prism, GraphPad Software, San Diego, CA, USA) to compare FE and IE for each crude xylan source. Asterisks indicate statistically significant differences between enzyme systems (*p < 0.005; ***p < 0.0005). Total XOS represents the sum of X2–X5. Conversion (%) was calculated as the percentage of total XOS produced relative to the initial crude xylan concentration

**Table 2:** Survival of *Artemia salina* exposed to xylooligosaccharides (XOS) produced from xylans derived from different agricultural residues by immobilized and free endo-xylanase

Source of xylan	Survival rate (%) – XOS produced by immobilized enzyme (IE)	Survival rate (%) – XOS produced by free enzyme (FE)
Corn straw	93.3 ± 11.5ᵃA	93.3 ± 11.5ᵃA
Corn cob	93.3 ± 11.5ᵃA	93.3 ± 11.5ᵃA
Sugarcane bagasse	60.0 ± 20.0ᵇA	73.3 ± 11.5ᵇA
Sorghum biomass	66.0 ± 11.5ᵇA	53.0 ± 23.1ᵇA

Values are expressed as mean ± standard deviation (n = 3). Different lowercase letters (a, b) indicate significant differences among XOS produced from different crude xylan sources according to Tukey’s test (p < 0.05). Uppercase letters indicate no significant differences between free and immobilized enzyme systems for the same substrate (ANOVA, p > 0.05). The control group (without XOS) showed 100% survival, was used as a reference, and was not included in the statistical analysis. *IE* immobilized enzyme; *FE* free enzyme

## Results and discussion

### Enzyme immobilization

The immobilization of *T. lanuginosus* PC7S1T endo-xylanase on magnetic chitosan beads cross-linked with glutaraldehyde was highly effective, resulting in an immobilization yield of 90.65%, an efficiency of 73.66%, and a recovered activity of 66.77%. These values significantly exceed those reported by Gracida et al. (2019) for xylanase immobilized on magnetic chitosan using genipin, which achieved a yield of only 22.84% and a recovered activity of 63.27%.

### Reusability and storage stability of immobilized endo-xylanase

**Fig. 1:**
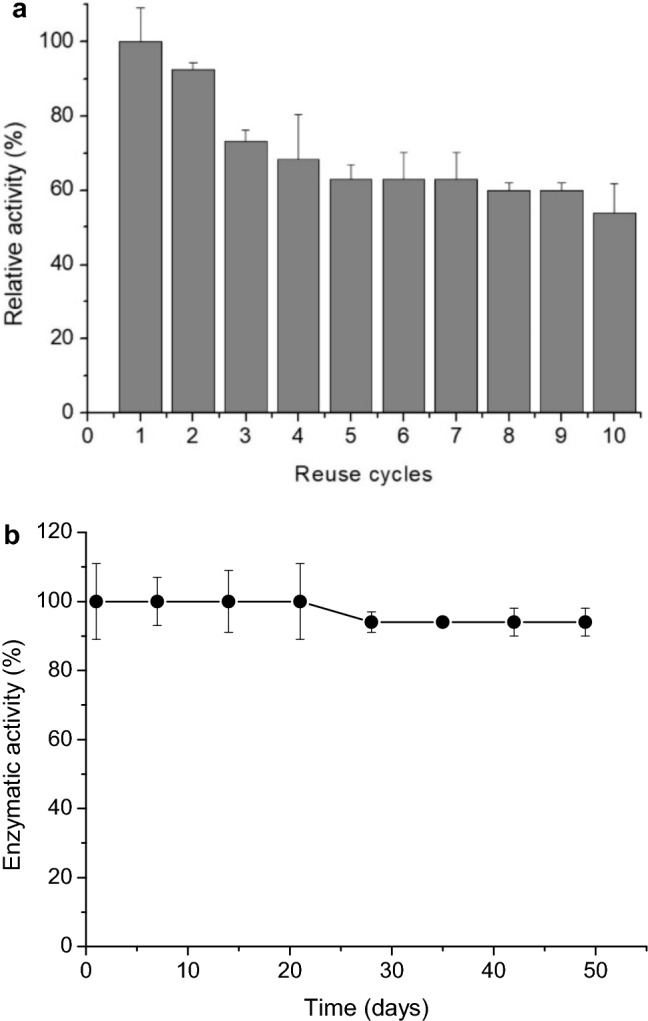
Reusability and storage stability of endo-xylanase immobilized on magnetic chitosan beads. (**a**) Reusability was evaluated over successive reaction cycles, with magnetic recovery of the biocatalyst between cycles, and expressed as residual activity (%) relative to the initial activity (100%) under standard assay conditions. (**b**) Storage stability was assessed by measuring residual activity (%) after storage at 4 °C for different time intervals, relative to the initial activity (100%). Data are presented as mean ± standard deviation (n = 2)

The immobilized endo-xylanase from *T. lanuginosus* PC7S1T on magnetic chitosan beads revealed strong operational stability, retaining 54% of its activity after 10 reaction cycles (Fig. 1a). This performance surpasses that of similar xylanases from the same species, including those immobilized on magnetic chitosan with genipin, which retained only 50% activity after three cycles (Gracida et al. 2019), and those entrapped in calcium alginate, which maintained 39% activity after five cycles (Nascimento et al. 2022).

These results indicate that magnetic chitosan cross-linked with glutaraldehyde is an effective and cost-efficient support for xylanase immobilization. Its combined properties, including biocompatibility, hydrophilicity, mechanical stability, and facile magnetic recovery, enhance enzyme reuse, while reducing activity loss as a result of leaching (Miri et al. 2024). Collectively, these characteristics support its potential for broader biotechnological applications (Kumari et al. 2018).

The immobilized enzyme also exhibited excellent storage stability, retaining 94% of its enzymatic activity after 50 d in distilled water at 4 °C, corresponding to only a 6% reduction in activity (Fig. 1b). Comparable findings were reported for XAn11–MNPs–E/N (XAn11-xylanase derived from *Aspergillus niger* and produced in *Pichia pastoris*), immobilized on magnetic nanoparticles, which retained 94% activity after 30 d at 4 °C (Salem et al. 2021). These results reinforce that immobilization enhances long-term stability at 4 °C, thus enhancing the enzyme suitability for industrial applications.

### Effect of temperature and thermal stability of immobilized endo-xylanase

**Fig. 2:**
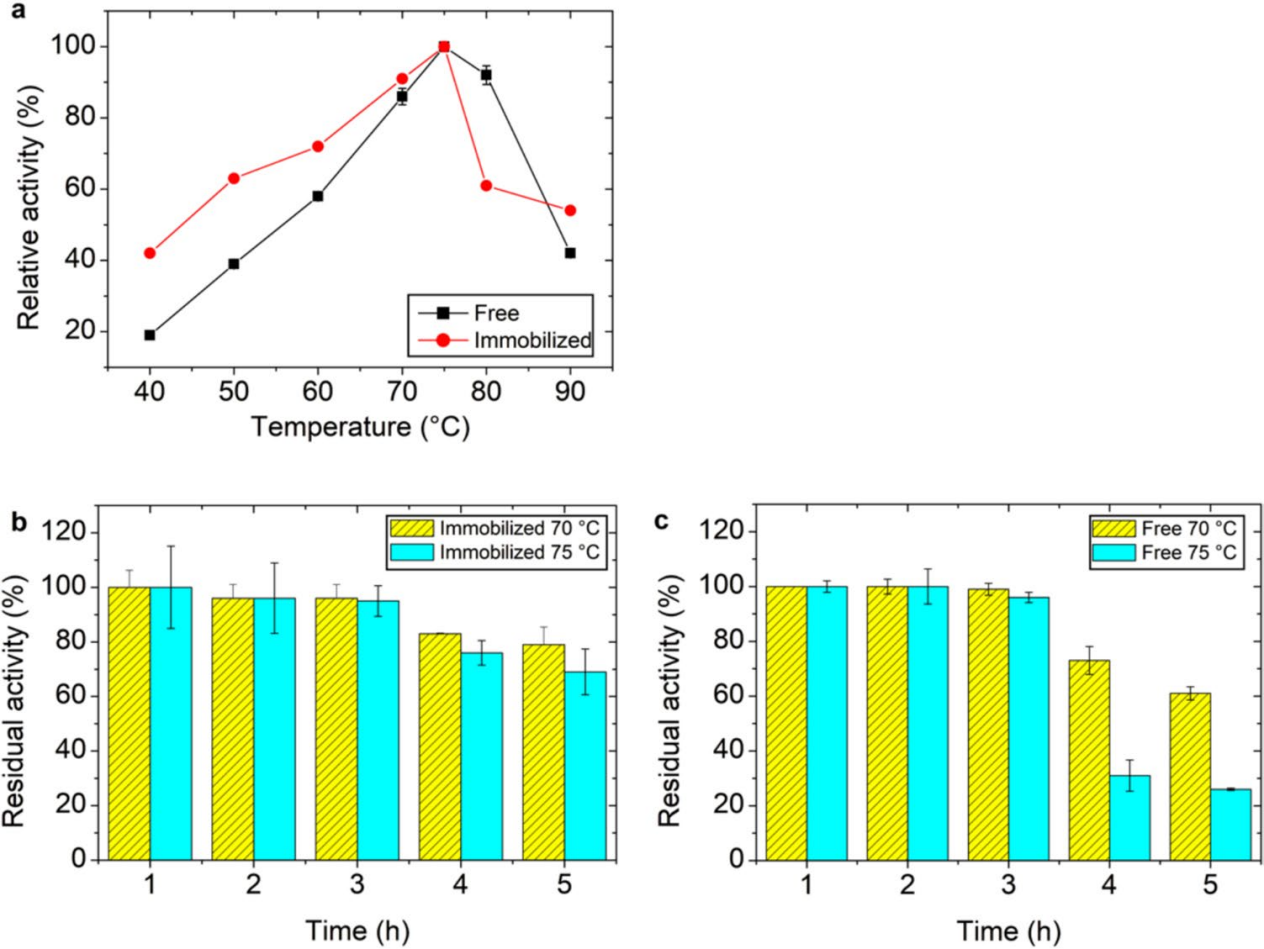
Effect of temperature on the catalytic performance and thermal stability of free and immobilized endo-xylanase. (**a**) Relative activity (%) of free and immobilized enzymes was determined as a function of temperature and expressed with respect to the maximum activity (100%) under the tested conditions. (**b**) Thermal stability of the immobilized enzyme was evaluated after pre-incubation at 70 °C and 75 °C for different time intervals and expressed as residual activity (%) relative to the initial activity before thermal treatment (100%). (**c**) Thermal stability of the free enzyme was determined under the same conditions. Data are presented as mean ± standard deviation (n = 2)

The activity of immobilized endo-xylanase was evaluated over 40–90 °C, with an optimum temperature of 75 °C observed for both free and immobilized forms (Fig. 2a). The absence of a shift in the optimal temperature suggests that immobilization did not significantly affect the enzyme’s catalytic microenvironment or active site conformation, as changes in this parameter are typically associated with structural or microenvironmental alterations (Mateo et al. 2007; Sheldon and van Pelt. 2013). This behavior may be attributed to the use of chitosan as a hydrophilic support, which helps preserve the native enzyme structure, as well as to covalent attachment through surface-exposed groups, minimizing structural perturbations and preventing interference with the active site. The optimum temperature observed here was higher than that reported for *T. lanuginosus* xylanase immobilized on magnetic chitosan via genipin cross-linking (70 °C) and on graphene oxide nanosheets (60 °C) (Mehnati-Najafabadi et al. 2018; Gracida et al. 2019). These differences likely reflect variations in immobilization strategies and support properties, which influence the enzyme microenvironment and thermal behavior.

Thermal stability assays (Fig. 2b–c) demonstrated that immobilization significantly enhanced enzyme resistance at elevated temperatures. After 5 h, the immobilized enzyme retained 79% and 69% of its initial activity at 70 and 75 °C, respectively, whereas the free enzyme retained 61% and 26% under the same conditions. This improved stability is commonly attributed to increased structural rigidity and reduced conformational mobility upon immobilization, which protect the enzyme against thermal denaturation (Mateo et al. 2007; Fernandez-Lafuente 2009).

### Effect of pH and stability of immobilized enzyme

**Fig. 3:**
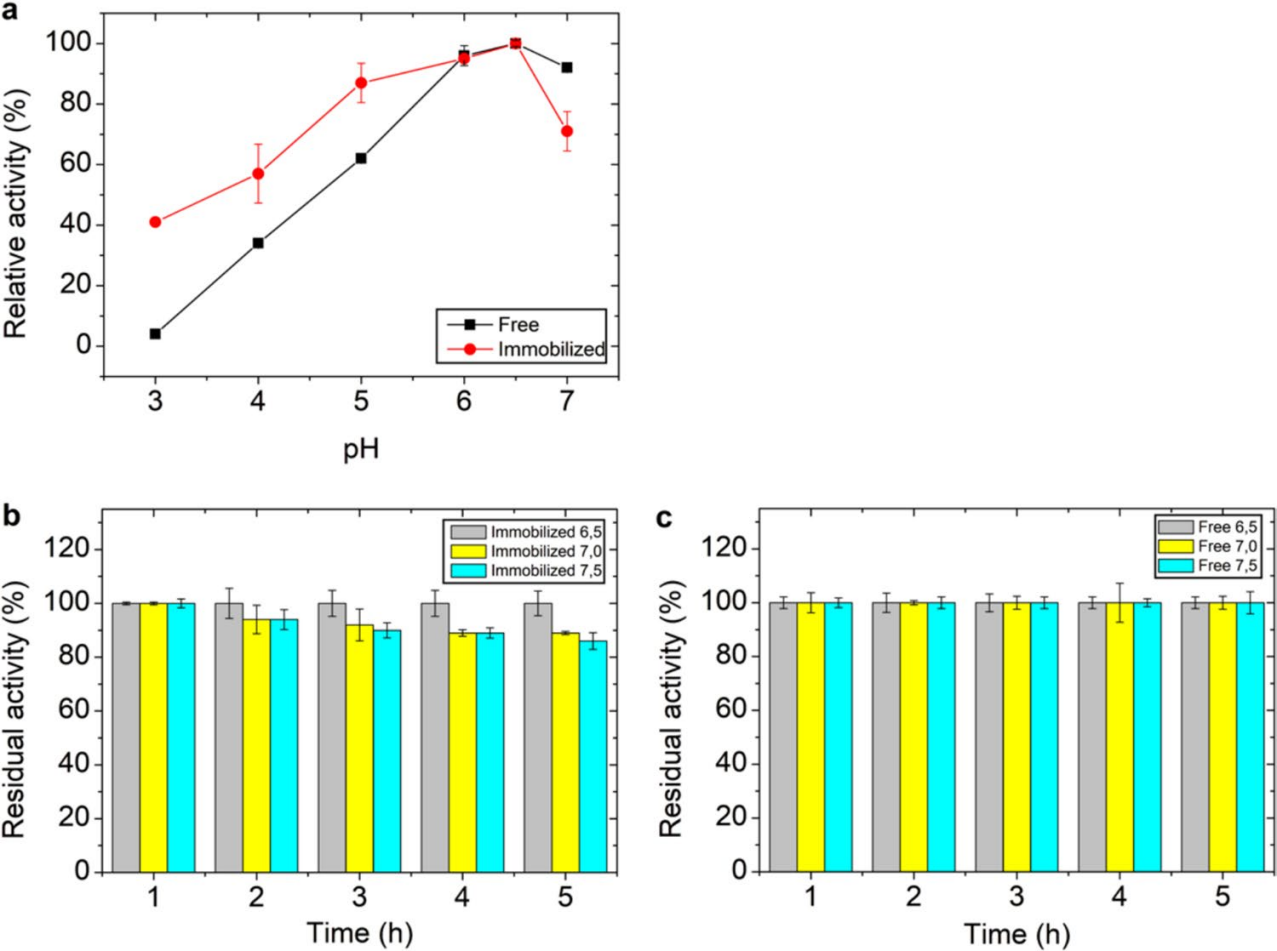
Effect of pH on the catalytic performance and pH stability of free and immobilized endo-xylanase. (**a**) Relative activity (%) of free and immobilized enzymes was determined at different pH values and expressed with respect to the maximum activity (100%) under the tested conditions. (**b**) pH stability of the immobilized enzyme was evaluated after incubation at pH 6.5, 7.0, and 7.5 for 5 h and expressed as residual activity (%) relative to the initial activity before incubation (100%). (**c**) pH stability of the free enzyme was determined under the same conditions. Data are presented as mean ± standard deviation (n = 2)

The influence of pH on the activity of immobilized endo-xylanase was evaluated at pH 3.0, 4.0, 5.0, 6.0, 6.5, 7.0, and 8.0 and compared with that of the free enzyme (Fig. 3a). Immobilization onto magnetic chitosan did not alter the optimal pH of the enzyme, which remained at 6.5 and was similar to that of the free enzyme. The absence of a shift in optimal pH indicates that the immobilization process preserved the ionization state of amino acid residues involved in catalysis, as changes in this parameter are typically associated with alterations in the enzyme microenvironment (Mateo et al. 2007; Sheldon and van Pelt. 2013). This preservation may be associated with the hydrophilic nature of the support and the immobilization strategy, which minimizes disturbances in the enzyme microenvironment and helps maintain the ionization state of catalytic residues. This behavior contrasts with that reported for *T. lanuginosus* xylanase immobilized on superparamagnetic graphene oxide nanosheets, where the optimal pH shifted from 6.5 (free enzyme) to 7.5 after immobilization (Mehnati-Najafabadi et al. 2018).

The immobilized endo-xylanase exhibited complete stability at the optimal pH of 6.5 over 5 h. However, under neutral (pH 7.0) and slightly alkaline (pH 7.5) conditions, activity reductions of 11% and 14%, respectively, were observed. In contrast, the free enzyme retained 100% activity under the same conditions (Figs. 3b and c). The lower pH stability of the immobilized enzyme at neutral and alkaline pH may be related to electrostatic interactions between the enzyme and the support surface, as well as residual charges introduced by glutaraldehyde during cross-linking. These interactions may induce subtle conformational or microenvironmental changes under non-optimal conditions, which can affect enzyme stability without significantly altering the optimal catalytic parameters (Bhushan et al. 2015).

### XOS production from crude xylan using free and immobilized endo-xylanase

Hydrolysis of crude xylan obtained from sugarcane bagasse, corn cob, corn straw, and sorghum biomass resulted in distinct XOS profiles depending on whether endo-xylanase was used in its free or immobilized form (Table 1). The free enzyme predominantly produced xylobiose (X2), representing 40%–41% of total products. In contrast, the immobilized enzyme favored oligosaccharides with a higher degree of polymerization, particularly xylotriose (X3) and xylotetraose (X4). For sugarcane bagasse, X3 and X4 accounted for 34% and 39% of total XOS, respectively, whereas in corn cob and corn straw, X3 represented 43–47% and X4 46%–53% of total products. Xylopentaose (X5, approximately 10%) was detected exclusively in reactions catalyzed by the immobilized biocatalyst.

No detectable xylose (X1) was observed in any reaction, indicating that β-xylosidase activity was not present in the enzyme preparation and confirming the typical endo-hydrolytic cleavage pattern of β-1,4-xylosidic bonds. This behavior is consistent with reports for *T. lanuginosus* PC7S1T xylanase, which predominantly generates X2–X4 without detectable xylose formation (Della Torre et al. 2023). Therefore, immobilization did not alter the intrinsic catalytic mechanism of the enzyme but modulated product selectivity.

The conversion of crude xylan into XOS ranged from 12–15% for the free enzyme and 7–11% for the immobilized derivative across the evaluated substrates (Table 1). Although immobilization moderately reduced overall conversion efficiency, it consistently shifted product distribution toward longer-chain XOS (X3–X5). This effect is likely related to diffusion limitations within the magnetic chitosan support, which may restrict secondary hydrolysis of intermediate oligosaccharides, as well as to microenvironmental stabilization of the immobilized enzyme (Rajagopalan et al. 2016; Nascimento et al. 2022).

From an application standpoint, the preferential accumulation of X3–X5 is advantageous because these fractions are associated with enhanced prebiotic functionality. When combined with magnetic recoverability and reusability, immobilization emerges as a promising strategy for controlled XOS production from lignocellulosic agricultural residues, enabling modulation of the product profile without compromising enzymatic integrity.

### SEM analysis of crude xylan from agricultural waste after treatment with immobilized endo-xylanase

**Fig. 4:**
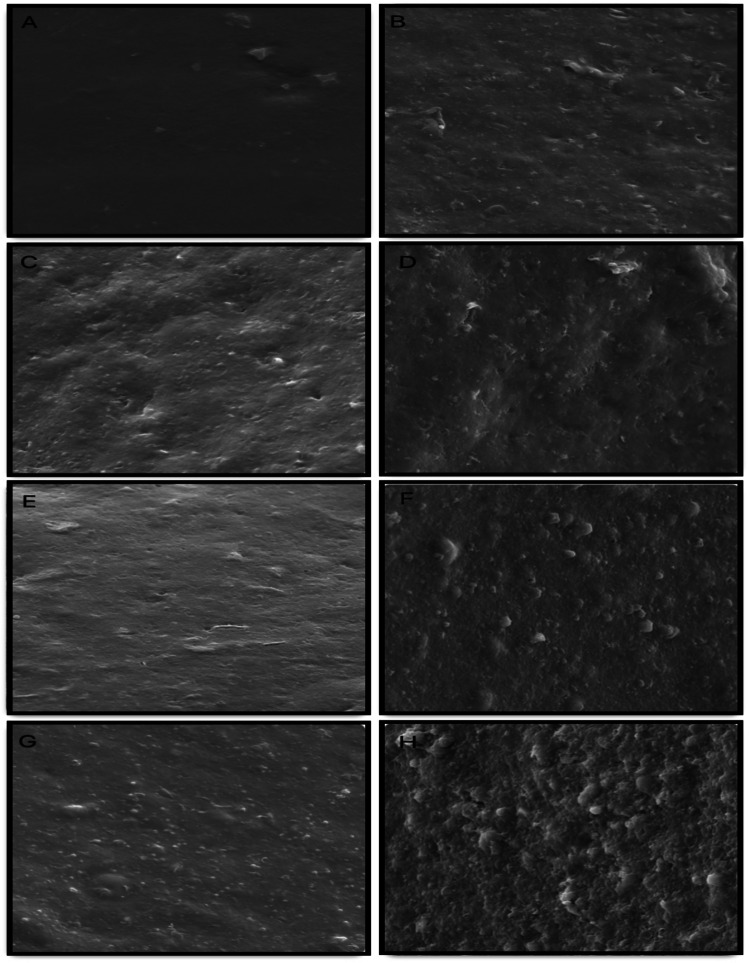
Scanning electron microscopy (SEM) images of xylan-rich fractions from agricultural residues before and after enzymatic hydrolysis with immobilized endo-xylanase. Samples were incubated with the immobilized enzyme at 65 °C for 48 h and observed at 5,000 × magnification. (**A**, **B**) Corn straw: control (**A**) and enzyme-treated (**B**). (**C**, **D**) Corn cob: control (**C**) and enzyme-treated (**D**). (**E**, **F**) Sugarcane bagasse: control (**E**) and enzyme-treated (**F**). (**G**, **H**) Sorghum biomass: control (**G**) and enzyme-treated (**H**)

SEM analysis revealed morphological alterations in crude xylan extracted from agricultural residues before and after treatment with immobilized endo-xylanase. Enzyme-treated samples exhibited rough and disorganized surfaces (Figs. 4B, D, F, and H), whereas untreated samples showed smooth and more organized structures (Figs. 4A, C, E, and G). These observations indicate effective depolymerization of the crude xylan. Similar structural disruption of wheat straw following alkaline pretreatment and enzymatic hydrolysis has been reported, resulting in increased surface area and fiber exposure compared with untreated controls, which maintain a uniform and compact morphology (Hamid et al. 2023).

### Toxicity analysis of XOS produced from crude xylan by free and immobilized endo-xylanase in *A. salina*

The toxicity of XOS was evaluated using *A. salina*, and no significant differences in survival rates were observed between hydrolysates produced by free and immobilized endo-xylanase for the same xylan source (Table 2). This indicates that the immobilization process using glutaraldehyde and magnetic chitosan did not introduce additional toxicity into the system.

Survival rates varied significantly depending on the xylan source used for XOS production (p < 0.05). XOS derived from corn straw and corn cob showed high survival rates (93.3%), indicating low toxicity. In contrast, XOS produced from sugarcane bagasse and sorghum biomass resulted in significantly lower survival rates (60.0–73.3% and 53.0–66.0%, respectively), suggesting the presence of inhibitory compounds. These effects may be associated with phenolic compounds released during biomass processing, such as vanillin, vanillic acid, and ferulic acid, which are known to exert toxic and inhibitory effects on aquatic organisms (Gufe et al. 2024).

These findings are consistent with previous reports describing the ecotoxic and inhibitory effects of phenolic compounds generated during the hydrolysis of lignocellulosic residues (Michelin et al. 2016; Gufe et al. 2024). The presence of such byproducts, particularly in hydrolysates derived from more recalcitrant or lignin-rich biomasses, highlights the need for strategies to reduce or remove these compounds. Overall, the ecotoxicological assessment using *A. salina* indicates that XOS toxicity is primarily influenced by the biomass source instead of the enzyme form.

### Industrial applicability and scalability considerations

Although a purified endo-xylanase was used in this study to enable a controlled assessment of catalytic performance and product selectivity, enzyme purification can increase production costs when processes are translated to industrial scale. In several industrial biocatalytic applications, partially purified or crude enzyme preparations are preferred because they reduce downstream processing requirements and associated costs (Sheldon and van Pelt. 2013). In this context, enzyme immobilization can represent an effective strategy to compensate for lower enzyme purity by improving operational stability and enabling catalyst reuse, which may reduce the effective cost per unit of XOS produced (Sheldon. 2007).

The magnetic chitosan-based immobilization system evaluated in the present study showed satisfactory storage stability and retained catalytic activity during repeated batch cycles. These characteristics indicate that the immobilized biocatalyst may be suitable for repeated-batch operations and potentially adaptable to semi-continuous processing systems. However, the long-term operational performance of the immobilized enzyme under continuous-flow conditions still requires further experimental investigation. From a process development perspective, successful scale-up will depend on several factors, including potential mass transfer limitations in heterogeneous catalytic systems, variability in lignocellulosic substrate composition, the economic feasibility of the support material, the efficiency of magnetic particle recovery, and the possible presence of inhibitory compounds generated during biomass pretreatment or hydrolysis (Michelin et al. 2016). Further investigation under continuous or pilot-scale conditions will be necessary to fully assess the practical applicability of this immobilized system.

## Conclusion

The immobilization of *T. lanuginosus* PC7S1T endo-xylanase on magnetic chitosan beads showed significant potential for the sustainable valorization of agricultural residues. The immobilized enzyme exhibited enhanced thermal stability, improved storage stability, and satisfactory operational reusability compared with the free enzyme, confirming the effectiveness of the support system. Notably, immobilization modulated product distribution, favoring the formation of xylotriose (X3) and xylotetraose (X4), oligosaccharides widely recognized for their prebiotic potential. Toxicity assessment revealed no detectable acute adverse effects when using the applied screening model, thus supporting the preliminary biosafety of the produced XOS.

Collectively, these findings reveal the technical feasibility of this immobilized biocatalyst for the selective production of value-added XOS from renewable biomass and provide a basis for future process optimization and scale-up evaluation.

## Data Availability

Data will be made available on request.

## Declarations

### Accession numbers

ITS sequence of the fungus *Thermomyces* lanuginosus PC7S1T deposited in the NCBI database under accession number KJ934703.1
